# Respiratory rate and its associations with disease and lifestyle factors in the general population – results from the KORA-FF4 study

**DOI:** 10.1371/journal.pone.0318502

**Published:** 2025-03-11

**Authors:** Ina-Maria Rückert-Eheberg, Alexander Steger, Alexander Müller, Birgit Linkohr, Petra Barthel, Melanie Maier, Julia Allescher, Moritz F. Sinner, Konstantinos D. Rizas, Wolfgang Rathmann, Karl-Ludwig Laugwitz, Stefan Kääb, Annette Peters, Georg Schmidt

**Affiliations:** 1 Institute of Epidemiology, Helmholtz Zentrum München, German Research Center for Environmental Health, Ingolstädter Landstraße, Munich-Neuherberg, Germany; 2 Department of Internal Medicine I, TUM School of Medicine and Health, Technical University of Munich, University Hospital, Munich, Germany,; 3 DZHK (German Centre for Cardiovascular Research), Partner Site Munich Heart Alliance, Munich, Germany,; 4 Department of Medicine I, LMU University Hospital LMU Munich, Munich, Germany,; 5 Institute for Biometrics and Epidemiology, German Diabetes Center, Leibniz Center for Diabetes Research, Heinrich Heine University Düsseldorf, Düsseldorf, Germany,; 6 German Center for Diabetes Research, München-Neuherberg, Germany,; 7 Institute for Medical Information Processing, Biometry and Epidemiology, Ludwig-Maximilians-Universität München, Munich, Germany; Shanghai Jiao Tong University School of Medicine, CHINA

## Abstract

**Objective:**

The aim of the study was to derive median age- and sex-specific respiratory rates in a population-based sample of adults and to identify disease and lifestyle factors associated with elevated respiratory rates.

**Methods:**

In the population-based KORA FF4 study conducted in Augsburg, Germany, 5-minute 12-lead resting electrocardiograms (ECGpro-system, AMEDTEC) were recorded in 2,224 participants from 39 to 88 years. Respiratory rate was derived from these electrocardiograms. Sex- and age-specific medians, IQRs, and percentiles were calculated. Associations of sociodemographic, disease, and lifestyle variables with elevated resting respiratory rate were assessed by univariable and multivariable logistic regression analyses.

**Results:**

Respiratory rate decreased slightly from youngest to middle-aged women and men and increased in old age. Overall, median (IQR) was 15.80 (3.16) breaths per minute (brpm). Five percent of the participants had values lower than 12.06 brpm, and five percent had values above 20.06 brpm (95^th^ percentile). Elevated respiratory rates of ≥  18.6 brpm were found in 13.8% (*n* =  308). In an adjusted logistic regression model, age, abdominal obesity, diabetes, COPD, smoking, and low education were significantly associated with elevated respiratory rate. Stratified analyses showed that education appeared to be more relevant in women, while the effect of diabetes was more pronounced in men.

**Conclusions:**

High respiratory rate may be an indicator of impaired health in the general population, especially regarding pulmonary and metabolic characteristics, and unfavorable lifestyle and living conditions. Individuals with an increased respiratory rate should therefore be examined and followed up more closely to recognize diseases and adverse progressions at an early stage and to possibly prevent them.

## Introduction

In the Book of Prognostics, written 400 B.C.E., Hippocrates emphasized the diagnostic importance of breathing: “Respiration, when frequent, indicates pain or inflammation in the parts above the diaphragm: a large respiration performed at a great interval announces delirium; but a cold respiration at nose or mouth is a very fatal symptom. Free respiration is to be looked upon as contributing much to the safety of the patient in all acute diseases, such as fevers, and those complaints which come to a crisis in forty days.” [1].

Today, it is generally recognized that, similar to heart rate, blood pressure, and body temperature, respiratory rate is a crucial vital sign to assess a person’s state of health as it varies acutely with exertion, stress, fever, and sepsis. According to the “Third International Consensus Definitions for Sepsis and Septic Shock”, the quick SOFA score including altered mentation, systolic blood pressure of 100 mm Hg or less, and respiratory rate of 22/min or greater, provides simple bedside criteria to identify adult patients with suspected infection who are likely to have poor outcomes [2].

Respiratory rate has been identified as an indicator of clinical deterioration and mortality in hospitalized patients [3–5] and older outpatients [6, 7] on the one hand. On the other hand, recent studies have shown that slow breathing or breathing techniques derived from Yoga, Zen or Qi Gong may be able to regulate blood pressure [8], increase cognitive function and reduce anxiety and stress [9, 10], alleviate pain [11], and improve symptoms of depressive disorder [12]. Nevertheless, respiratory rate has often been disregarded [13] or regretfully termed ‘neglected vital sign’ [14] in the past and it is still unclear how to react if the wearable or the monitor in the clinic indicates an increased respiratory rate, what the expected frequency spectrum is, and if respiratory rate indicates existing, previously undiagnosed comorbidities. These questions require evidence from large observational studies.

A description of sex- and age-specific normal values is needed to estimate the severity of respiratory diseases such as chronic obstructive pulmonary disease (COPD) and pneumonia, to define fitness levels, and to describe tachypnoea [15]. To date, there is no firm evidence on the distribution of resting respiratory rate in the general adult population. A study in non-institutionalized elderly participants in Spain found that the 2.5–97.5-percentiles were 12.0–28.2 breaths per minute (brpm) in those aged>=65 years and 9.8–30.0 brpm in those aged>=80 years [16]. Evidence-based normal values have not been defined so far and guidelines are based on clinical consensus only.

Autonomic functionality and interactions of the autonomic nervous system with the cardiovascular and respiratory systems can be quantified by ECG-derived autonomic parameters, such as respiratory rate, heart rate and deceleration capacity, a heart rate variability parameter reflecting the vagally mediated regulation of the heart rate. Based on these parameters, the prognosis of patients with cardiovascular diseases can be estimated fairly well [17–20]. In a study including patients who survived acute myocardial infarction, a Polyscore of autonomic parameters derived from ECG, blood pressure, and respiration strongly predicted cardiac and all-cause mortality and was able to identify patients with a very low mortality risk [21]. Moreover, there is some evidence on the prognostic utility of these parameters in the general population [22]. It is still crucial to analyze the individual components of such scores in more detail.

Little is known about the correlations of respiratory rate with sex and age, life-style related risk factors, and clinical variables. Baumert *et al*. conducted an observational study on the association of nocturnal mean respiratory rate and mortality among community-dwelling older men and women. They found that in men, respiratory rate increased with age, body mass index (BMI), and systolic blood pressure and was higher in those with diabetes, asthma or COPD, smokers, and those with lower alcohol consumption. All associations were statistically non-significant in women; however, women were underrepresented in this cohort [7]. Furthermore, it has been found that respiratory rates are shaped by genetic, physiological, and environmental factors. In particular, high-a[ltitude environments expose individuals to hypoxia, leading to adaptations that affect breathing patterns [23].

The objective of this study was to derive the resting respiratory rate from 5-minute 12-lead electrocardiograms in a population-based study in Southern Germany and examine associations between increased respiratory rate and socioeconomic, lifestyle, and clinical factors. In addition, we assessed its correlation with heart rate, since the respiratory and cardiovascular systems are closely linked via autonomous interactions and reflexes. The two parameters do not depict the same mechanisms but are often altered concordantly.

## Methods

### KORA study and participant selection

KORA (Cooperative Health Research in the Region of Augsburg) is a population-based health cohort in the city of Augsburg and two surrounding counties

Augsburg and Aichach-Friedberg, in Southern Germany [24]. Briefly, the participants were randomly selected in a sex- and age-stratified manner from population registries from all inhabitants with main place of residence in the study region and German nationality in four cross-sectional surveys from 1984–2001. In 1999–2001, 4,261 participants from 25 to 74 years were enrolled in the KORA S4 study.

**Fig 1:**
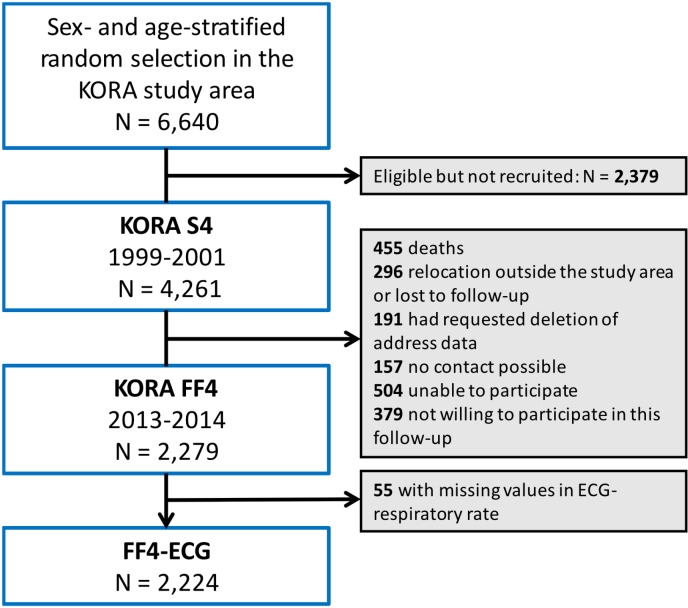
Flow chart of the study population.

The KORA FF4 study is the second follow-up of the KORA S4 study conducted from 03.06.2013 to 27.09.2014. Participants of KORA S4 were considered ineligible for FF4 if they died (n = 455, 10.7%), lived too far outside the study region or were completely lost to follow-up (n = 296, 6.9%), or had requested deletion of their address data (n = 191, 4.5%). Of the remaining 3,319 eligible persons, 157 could not be contacted, 504 were unable to attend the study centre, and 379 were not willing to participate in this follow-up, resulting in a response rate of 68.7% and a FF4 study population of 2,279 participants [25] (Fig 1).

The study was carried out in accordance with the Declaration of Helsinki. This included written informed consent from all participants and approval of the surveys by the ethics committee of the Bavarian Chamber of Physicians, Munich (FF4 EC No. 06068).

### Electrocardiogram recoding

A 5-minute 12-lead resting electrocardiogram (ECGpro-system, Fa. AMEDTEC) was recorded in all participants. The electrocardiograms were digitally stored and semi-automatically analyzed. After initial algorithm-based processing, the electrocardiograms were reviewed by experienced professionals. If necessary, artefacts were removed and QRS classifications were corrected manually to obtain an accurate sequence of heartbeat intervals.

Respiratory rate was indirectly calculated from the electrocardiogram tracings as previously described [26]. Respiratory activity influences oscillations of beat-to-beat intervals, amplitudes of QRS complexes, and QRS vector angles and magnitudes. Time series of these parameters were derived. For each time series, all local maxima were identified and the mean interval duration between consecutive local maxima was determined. Respiratory rate was then calculated from the median of these reciprocal interval durations [26]. According to previous studies, elevated respiratory rate was prespecified at more than or equal to 18.6 breaths per minute (brpm) [26, 27]. This was equivalent to the 86^th^ percentile in the data used here.

Of the 2,279 participants in FF4, we excluded those with missing data on respiratory rate leaving *n* =  2,224 participants.

### Covariates

At the KORA study center, an interview, physical examination, and a blood withdrawal were performed by trained staff. Self-reported information on the demographic variables sex, age, and living alone or with a partner, socioeconomic factors such as school education (low/secondary school, middle/secondary school leaving certificate, high/A-levels) reported at baseline and lifestyle factors including smoking (never smoker, ex-smoker, (occasional and regular) smoker), and physical activity (based on leisure activity during summer and winter (regularly > 2 hours/week, regularly about 1 hour/week, irregularly about 1 hour/week, almost none or none)) [28] were collected in a standardized computer-assisted personalized interview. Alcohol consumption was assessed using a validated recall method in which participants were asked how much beer, wine, and spirits they consumed on the previous weekday and weekend [29]. No alcohol consumption was defined as 0 g/day, moderate alcohol consumption as 0.1–39.9 g/day for men and 0.1–19.9 g/day for women and heavy alcohol consumption as ≥  40 g/day for men and ≥  20 g/day for women. The participants were asked whether they’ve had a myocardial infarction, a stroke, cancer, a diagnosis of chronic obstructive pulmonary disease (COPD) or of asthma. Depressive symptoms were assessed by the Brief Patient Health Questionnaire (PHQ-D short version). Current hypertension was defined as blood pressure of ≥  140/90 mmHg or known hypertension treated with medications. Waist circumference (WC) was measured to the nearest 0.1 cm at the midpoint between the lower margin of the least palpable rib and the top of the iliac crest with stretch-resistant tapes. Abdominal obesity was defined as a WC of > 88 cm in women and > 102 cm in men [30]. Diabetes status was assessed by an oral glucose tolerance test (OGTT) that was performed in all participants without known diabetes as described previously [31] (S1 Table).

### Statistical analyses

In subgroups of sex and age, respiratory rate and heart rate were analyzed by computation of medians, interquartile ranges, and percentiles. QQ-plots, Shapiro-Wilk- and Kolmogorov-Smirnov tests were used to check for normal distributions and Kendall’s rank correlation tau as well as Spearman’s rho were applied to analyze the correlation of the two variables.

Participant characteristics were compared between subgroups with normal and elevated respiratory rates using univariable logistic regression models. Adjusted odds ratios with 95% - confidence intervals were obtained from multivariable logistic regression models. Biological and clinical plausibility and the avoidance of multicollinearity were main criteria for the selection of the covariables. Multicollinearity was checked by calculating the variance inflation factor (VIF) and variables with values >  5 were excluded from the model. Significance was assumed for a two-sided p < 0.05. All statistical analyses were performed using R4.2.1 and R-Studio (Boston, MA, USA).

## Results

### Study population

The study population included 2,224 participants (1,069 men and 1,155 women) of the KORA FF4 study with complete data on respiratory rate. The age ranges in both sexes were 39 to 88 years (mean age in men 61.1 ± 12.6 years and in women 60.1 ± 12.1 years).

### Respiratory rates in the whole study population and by sex and age

In all 2,224 participants, respiratory rate at rest ranged from a minimum of 9.19 brpm to a maximum of 28.17 brpm. Median and IQR were 15.80 brpm and 3.16 brpm, respectively. In participants 39 to 48-years-old, median respiratory rate was 15.62 brpm, 15.26 brpm in those from 49 to 58 years-old, 15.80 brpm in those from 59 to 68 years-old, 16.15 brpm in those from 69 to 78 years-old, and 17.01 brpm in those from 79 to 88 years-old. Five percent of the participants had values lower than 12.06 brpm (5^th^ percentile) and 5% of participants had values above 20.06 brpm (95^th^ percentile). In 13.8% (*n* =  308) of the participants, resting respiratory rate was higher than or equal to 18.6 brpm. The percentages of participants with an elevated respiratory rate of ≥  18.6 brpm were 11.7% in those at ages 39 to 48 years, 9.6% in individuals at ages 49 to 58 years, 14.0% in those at ages 59 to 68 years, 16.2% in individuals at ages 69 to 78 years, and 24.9% in those between the ages of 79 to 88 years.

Age dependency was observed in both sexes. In men, mean respiratory rate was slightly higher in participants at ages 39 to 48 years (15.37 brpm) than in those from 49 to 58 years-old (15.16 brpm) and highest in the older age groups (e.g., 16.91 brpm in participants at ages 79 to 88 years). Consistently in women, median respiratory rate was 15.80 brpm in the youngest age group, 15.33 brpm in the second youngest age group, and 17.07 brpm in the oldest age group (Table 1).

**Table 1:** Respiratory rate (breaths per minute, brpm) by age and sex.

Age	Sex	N	Min	Max	Median	IQR	Quantile 5	Quantile 25	Quantile 75	Quantile 95	Respiratory rate ≥ 18.6 brpm
39–48 y.	Men	221	10.31	28.17	15.37	3.51	11.35	13.46	16.97	19.25	12.2%
	Women	251	9.19	24.53	15.80	2.77	12.09	14.28	17.05	19.55	11.2%
49–58 y.	Men	254	9.47	21.94	15.16	3.16	11.57	13.69	16.85	19.88	10.2%
	Women	288	9.50	21.12	15.33	2.79	11.79	14.12	16.91	19.72	9.0%
59–68 y.	Men	254	10.67	22.26	15.68	3.03	12.28	14.14	17.17	20.29	13.0%
	Women	302	10.22	24.56	15.92	3.05	12.26	14.53	17.58	19.81	14.9%
69–78 y.	Men	234	9.92	22.25	16.26	3.17	12.76	14.62	17.79	20.41	17.9%
	Women	223	10.54	22.33	16.06	2.87	12.82	14.79	17.66	19.92	14.3%
79–88 y.	Men	106	10.45	22.59	16.91	3.03	12.88	15.46	18.49	20.76	24.5%
	Women	91	11.99	22.93	17.07	3.44	13.88	15.14	18.58	21.16	25.3%

**Fig 2:**
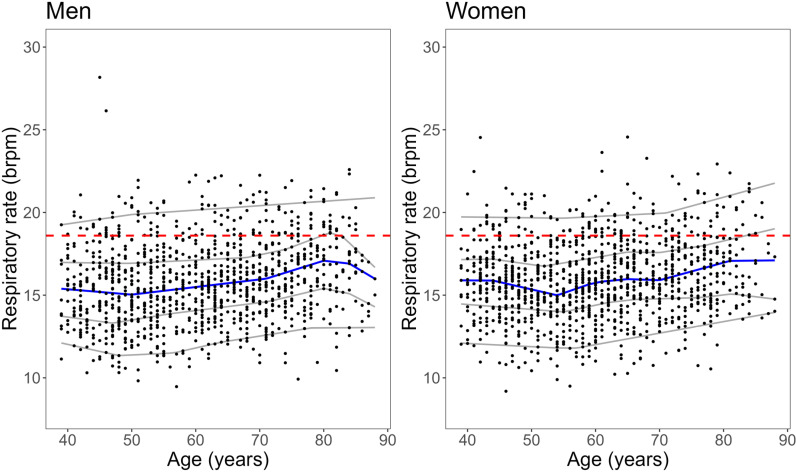
Scatterplots of respiratory rate and age by sex.

**Fig 3:**
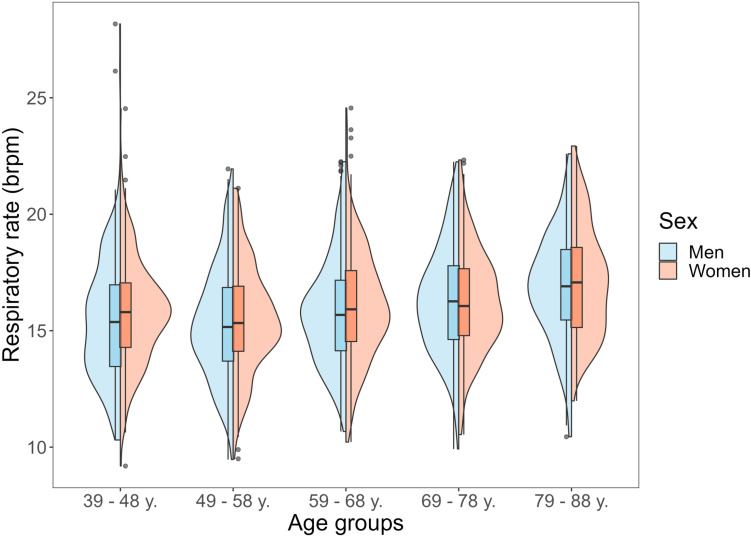
Violin-boxplot of respiratory rate by age and sex.

Respiratory rate was approximately normally distributed, and most outliers were found in the youngest age group (Figs 2 and 3).

Dashed red line indicates cut point of 18.6 brpm for elevated values; blue (median) and grey lines (from bottom to top: 5^th^, 25^th^, 75^th^, 95^th^-percentiles) derived by quantile regression.

### Associations of elevated respiratory rate with disease and lifestyle factors

Various clinical and lifestyle factors differed between participants with normal versus elevated respiratory rates (S2 Table). Individuals with elevated respiratory rate were more often in the three oldest age groups. They had more comorbidities such as a history of stroke, obesity, arterial hypertension, and chronic obstructive pulmonary disease. Additionally, they were less physically active, had a lower school education, lived alone more often, and had more depressive symptoms.

Table 2 is a regression table and shows frequencies and percentages of normal vs. elevated respiratory rates in groups of individuals with certain disease and lifestyle characteristics, including univariable regression results. Univariable ORs for a high respiratory rate were significant in the two oldest age groups (vs. the youngest age group), the lowest (vs. the highest) school education, abdominal obesity, physical inactivity, smoking (vs. never-smoking), living without a partner, previous stroke, depressive symptoms, hypertension, COPD, (pre)-diabetes, and heart rate. A protective effect in the univariable model was found for a medium consumption of alcohol (less than 40g/day in men and less than 20g/day in women vs. no consumption).

**Table 2:** Regression table – univariable associations of sociodemographic, disease and lifestyle factors with elevated respiratory rate.

		< 18.6 brpm *N* = 1,916 (86.2%)	≥ 18.6 brpm (elevated) *N* = 308 (13.8%)	Univariable OR (95%-CI, p)
Sex	Women	1001 (86.7)	154 (13.3)	–
	Men	915 (85.6)	154 (14.4)	1.09 (0.86–1.39, p = 0.464)
Age	39–48 years	417 (88.3)	55 (11.7)	–
	49–58 years	490 (90.4)	52 (9.6)	0.80 (0.54–1.20, p = 0.288)
	59–68 years	478 (86.0)	78 (14.0)	1.24 (0.86–1.80, p = 0.259)
	69–78 years	383 (83.8)	74 (16.2)	1.46 (1.01–2.14, p = 0.046)
	79–88 years	148 (75.1)	49 (24.9)	2.51 (1.63–3.85, p < 0.001)
School education	Highest	519 (91.2)	50 (8.8)	–
	Middle	509 (89.0)	63 (11.0)	1.28 (0.87–1.91, p = 0.209)
	Lowest	883 (81.9)	195 (18.1)	2.29 (1.66–3.21, p < 0.001)
Living alone	Lives with partner	1500 (87.4)	216 (12.6)	–
	Lives without partner	416 (81.9)	92 (18.1)	1.54 (1.17–2.00, p = 0.002)
Waist circumference	≤ 88 cm in men, ≤ 102 cm in women	987 (92.0)	86 (8.0)	–
	> 88 cm in men, > 102 cm in women	928 (80.8)	220 (19.2)	2.72 (2.10–3.56, p < 0.001)
Physical activity	Active	1134 (89.1)	139 (10.9)	–
	Inactive	782 (82.2)	169 (17.8)	1.76 (1.38–2.25, p < 0.001)
Smoking	Never-smoker	807 (87.1)	119 (12.9)	–
	Ex-smoker	826 (86.4)	130 (13.6)	1.07 (0.82–1.39, p = 0.632)
	Smoker	283 (82.7)	59 (17.3)	1.41 (1.00–1.98, p = 0.046)
Alcohol	No consumption	507 (83.5)	100 (16.5)	–
consumption	Less than 40g/day in men, 20g/day in women	1057 (87.7)	148 (12.3)	0.71 (0.54–0.94, p = 0.015)
	40g/day in men or more, 20g/day in women or more	351 (85.4)	60 (14.6)	0.87 (0.61–1.22, p = 0.420)
Hypertension	No current hypertension	1201 (88.6)	154 (11.4)	–
	Current hypertension	711 (82.3)	153 (17.7)	1.68 (1.32–2.14, p < 0.001)
Prior Myocardial	No MI	1853 (86.4)	292 (13.6)	–
infarction	MI	58 (78.4)	16 (21.6)	1.75 (0.96–3.01, p = 0.053)
Prior Stroke	No stroke	1874 (86.6)	290 (13.4)	–
	Stroke	41 (71.9)	16 (28.1)	2.52 (1.36–4.47, p = 0.002)
Cancer	Never cancer	1705 (86.3)	271 (13.7)	–
	Cancer	211 (85.1)	37 (14.9)	1.10 (0.75–1.58, p = 0.605)
Depressive	No or little depressive sympt.	1840 (86.5)	286 (13.5)	–
symptoms	Depressive symptoms	74 (77.9)	21 (22.1)	1.83 (1.08–2.96, p = 0.018)
COPD	No COPD	1786 (87.2)	263 (12.8)	–
	COPD	124 (74.3)	43 (25.7)	2.35 (1.61–3.38, p < 0.001)
Asthma	No asthma	1743 (86.2)	279 (13.8)	–
	Asthma	171 (86.4)	27 (13.6)	0.99 (0.63–1.48, p = 0.950)
Diabetes mellitus	Normal glucose tolerance	1287 (90.1)	142 (9.9)	–
(OGTT)	Prediabetes	319 (82.0)	70 (18.0)	1.99 (1.45–2.71, p < 0.001)
	Known or new diabetes	239 (74.9)	80 (25.1)	3.03 (2.23–4.11, p < 0.001)
	Unclear	71 (81.6)	16 (18.4)	2.04 (1.12–3.52, p = 0.014)
Heart rate	Mean (SD)	64.4 (8.9)	74.1 (12.4)	1.10 (1.08–1.11, p < 0.001)
	Median (IQR)	64.2 (11.4)	73.9 (16.1)	

COPD: chronic obstructive pulmonary disease; MI: myocardial infarction; OGTT: results of the oral glucose tolerance test

#### Multivariable logistic regression model.

**Fig 4:**
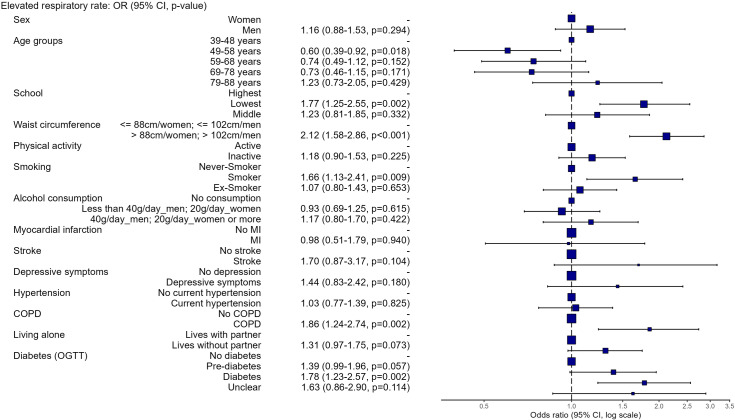
Multivariable logistic regression – Association of various disease and lifestyle factors with elevated respiratory rate (*N*=  2,194 participants with complete data).

In a multivariable logistic regression model (Fig 4) including 2,194 participants with complete data, elevated respiratory rate was not associated with sex. Participants at the age of 49–58 years had lower odds for an elevated respiratory rate than those in the youngest age group (39–48 years) (OR: 0.60, 95%-CI 0.39–0.92), while the odds in those older than 58 years were not significantly different from the odds in the youngest age group. Low education (OR: 1.77, CI: 1.25–2.55), abdominal obesity (OR: 2.12, CI: 1.58–2.86), diabetes (OR: 1.78, CI: 1.23–2.57), and pre-diabetes (OR: 1.39, CI: 0.99–1.96), smoking (OR: 1.66, CI: 1.13–2.41), and COPD (OR: 1.86, CI: 1.24–2.74) were significantly associated with elevated respiratory rate. Physical activity, alcohol consumption, living alone, myocardial infarction, stroke, depressive symptoms, and hypertension did not reach statistical significance in the adjusted analysis. Multicollinearity was ruled out since all VIF-values were <  2.

Models stratified by sex (S1 and S2 Figs) showed that the lowest school education (*n* =  549 (47.5%) in women; *n* =  529 (49.5%) in men) remained significant in women (OR: 2.29, CI: 1.32–4.17), but not in men (OR: 1.49, CI: 0.94–2.40), while diabetes (*n* =  134 (11.6%) in women; *n* =  185 (17.3%) in men) and pre-diabetes might play a bigger role in men than in women (OR for diabetes in women: 1.69, CI: 0.98–2.89; OR for diabetes in men: 1.82, CI: 1.08–3.03). However, the inclusion of interaction terms in the combined model revealed non-significant effects, suggesting that these differences may be due to chance or insufficient power.

In a further sensitivity analysis excluding 167 participants with COPD, pre-diabetes was significantly associated with elevated respiratory rate (OR: 1.50, CI: 1.03–2.15) and the odds of the second oldest age group (49–58 years) compared to the youngest age group was not significant anymore. The other factors remained similar (S3 Fig.)

### Characteristics of study participants with extreme respiratory rate measurements

Of 2,224 total study participants, 116 had respiratory rate values above their sex- and age-specific 95^th^-percentiles. 75.9% of them had abdominal obesity; 27.6% had diabetes, 22.4% had pre-diabetes, 26.7% were smokers, 37.9% were ex-smokers, 64.7% were physically inactive, 12.1% had COPD, and 69.0% had the lowest school education. Only two out of these 116 participants (2.6%) did not have any of the mentioned risk factors. One of them suffered from asthma and the other had no relevant comorbidity.

In comparison, of the 2,108 participants who had respiratory rates equal to or below their sex- and age-specific 95^th^-percentiles, 50.3% had abdominal obesity, 13.6% had diabetes, 17.2% had pre-diabetes, 14.8% were smokers, 43.3% were ex-smokers, 41.6% were physically inactive, 7.3% had COPD, and 47.3% had the lowest school education. In 162 (7.7%) of these individuals, none of the risk factors mentioned above including asthma were present.

### Correlation of respiratory rate and heart rate

Heart rate was rather stable across age groups and was higher in women than in men (S3 Table). The median heart rate in women was 65.95 beats per minute (bpm, IQR =  11.38) and 63.91 bpm (IQR =  13.38) in men.

Respiratory rate was positively correlated with heart rate (Kendall’s tau: 0.253, p <  0.001; Spearman’s rho: 0.368, p <  0.001). The median heart rate was 64.20 bpm (IQR =  11.35) in participants with normal and 73.91 bpm (IQR =  16.10) in those with elevated respiratory rates. The 95^th^-percentile for those with normal respiratory rate was at 79.56 bpm and at 92.41 bpm in those with elevated respiratory rate.

## Discussion

To our knowledge, this study describes ECG-derived respiratory rate in a large sample of the general population for the first time. While normal values of respiratory rate have been analyzed in children up to the age of 18 years, there are no population-based references in adults to date. According to 20 observational studies including 3,881 children, Fleming *et al*. [32] proposed median cut-offs of 43 brpm for newborn to three months old infants, 28 brpm for children aged two to three years, 19 brpm for children aged eight to twelve, 18 brpm for children aged twelve to 15, and 16 brpm for those aged 15 to 18 years. In the KORA FF4 study, median respiratory rate was 16 brpm in participants 39 to 48 years, 15 brpm in those 49 to 58 years, 16 brpm in those 59 to 68 years, 16 brpm in those 69 to 78 years, and 17 brpm in those 79 to 88 years old.

308 participants (13.8%) had an elevated respiratory rate ≥ 18.6 brpm. The odds of an elevated respiratory rate were higher with increasing age, with the presence of metabolic, cardiovascular, and pulmonary comorbidities, with low physical activity, active smoking, and with socioeconomic and psychosocial factors such as low school education, depression, and living without a partner. In a multivariable regression model, the following factors were independently associated with an increased respiratory rate:

### Abdominal obesity

The results of our study confirm that individuals with obesity (especially abdominal obesity) have higher respiratory rates. This is probably due to reduced tidal volumes and pulmonary restriction caused by the distribution of fat especially in the upper body. Thus, using waist circumference instead of BMI as an indicator of abdominal obesity is a reasonable and recommended alternative [33]. As summarized in a review by Littleton, several studies showed that pulmonary ventilation including total lung capacity, expiratory reserve volume, and functional residual capacity are affected by obesity. Interestingly, most of the effects of obesity on respiration are reversible by weight loss, which indicates that they may be caused by obesity itself [34].

### Diabetes

Known and newly diagnosed diabetes as well as pre-diabetes determined by an OGTT were associated with elevated respiratory rate, especially in men. An impaired lung function in patients with diabetes has been described in several reviews [35–37]. Obesity and cardiovascular diseases are frequent comorbidities in individuals with diabetes, which is why these factors were included as covariates in our models, however, diabetes itself appears to affect respiration. As outlined by Lecube *et al*., it has a damaging effect on the lung due to insulin and leptin resistance, chronic inflammation, autonomic neuropathy, and microvascular impairments. In the process, alveolar epithelia get thicker, the alveolar space is reduced, there are higher degrees of fibrosis, and a higher risk of sleep apnea. The authors recommend considering patients with diabetes as a vulnerable group for pulmonary dysfunction [38]. Interestingly, there is an ongoing discussion on whether reduced lung function may be a risk factor for incident diabetes as well [35,39]. Choi *et al*. conducted a longitudinal study in 3,864 adults in Korea and reported that lower forced vital capacity and lower forced expiratory volume predicted the onset of diabetes independently of many potential covariates and in a graded fashion [40]. Another current research question regards strength and function of the respiratory muscles: Schein *et al*. hypothesized that inspiratory muscle exercise as an alternative to conventional physical activity might lower glucose levels in patients with diabetes. In a small, randomized trial, they found that this type of exercise induced cardiovascular changes, however, it failed to improve glucose, glucose variability and autonomic control in their experimental setting [41].

### Smoking and COPD

In our adjusted regression model, we found significant associations of smoking and COPD with elevated respiratory rate. Smoking is a known causal risk factor of COPD and both smoking and COPD impair the pulmonary system [42]. Specifically, they lead to small airway narrowing and lower gas exchange rates [43] and, intuitively, they often result in higher respiratory rates to keep up the pulmonary gas exchange. Furthermore, toxins in cigarette smoke irritate the airways; activation of the sympathetic nervous system due to nicotine stimulation, chronic inflammation and swelling, mucus overproduction, cilia dysfunction, and alveolar damage among other factors may contribute to an elevated respiratory rate [44–46].

### School education

Even after adjustment for various confounding factors, low school education was still clearly associated with elevated respiratory rate. This observation indicates that further factors associated with low socio-economic status, such as disadvantageous living conditions at the place of residence (e.g., air pollution and noise) and unfavorable life circumstances (e.g., stress, unhealthy nutrition, sleep deprivation) may play a role [47]. Moreover, people with lower education are more often exposed to unhealthy working environments (e.g., to dust, chemicals, and vapors in the construction industry, agriculture, or production) and might have experienced more detrimental influences during gestation and childhood (e.g., exposure to passive smoke, less healthy nutrition) [48].

Our study reconciles disease, lifestyle, and socioeconomic factors associated with respiratory rate and indicates that the vital sign has remarkable diagnostic potential. It no longer requires special sensor technology and is easy to derive from ECGs in the clinical and ambulatory setting as well as from wearables. Thus, people with an increased respiratory rate should be examined and followed up more closely to detect and possibly prevent diseases and unfavorable progressions at an early stage.

### Strengths and limitations

A strength of the KORA study is its large sample size from the general population with rich phenotyping including ECG measurements. Moreover, the data were collected in a highly standardized manner. Breathing is very subjective, so measuring the respiratory rate while the patient or subject knows what is being measured can lead to altered results. One way to address this problem would be to take longer measurements and delete the first few minutes of them. However, this method is cumbersome and difficult to scale. Therefore, using ECG data is a very elegant way to determine respiratory rate without the described bias. Moreover, ECGs are universally available, scalable, the respiratory rate can be determined from any recorded ECG data set, and the standardized measurement can be easily reproduced.

There are certain limitations to our work as well: The study-design was cross-sectional and based on the second follow-up approximately 13 years after baseline, which might have introduced some selection bias. The results of our study may not be generalizable to other ethnicities or populations with different demographic or different health profiles. Lifestyle and disease records relied on self-report by the participants. Information on cardiac insufficiency, a condition that may also be associated with increased respiratory rate, was not available. Our results are adjusted for numerous known confounders, however, there might be additional unrecognized effects. Finally, participants were asked to take shallow breaths during the recording of the ECG, which might have influenced respiratory frequency in some individuals.

## Conclusions

Respiratory rate is an age-dependent vital sign. It is easy to measure and can be calculated indirectly from electrocardiogram recordings, avoiding distortion by the person’s awareness and voluntary control of breathing.

Increased respiratory rate is independently associated with obesity, diabetes, smoking, COPD, and low school education. It appears to be an indicator for impaired health especially regarding pulmonary and metabolic characteristics, and for unfavorable lifestyle and living conditions. To further confirm the predictive value of elevated respiratory rate on morbidity and mortality, studies with longer observation periods are needed.

## Supporting information

S1 File SupportingInfo_RespiratoryRate_PlosOne_Rueckert. (PDF)

## Abbreviations

BMI,: Body mass index;
KORA,: Cooperative Health Research in the Region of Augsburg; bpm, Beats per minute;
OGTT,: Oral glucose tolerance test;
brpm,: Breaths per minute;
VIF,: Variance inflation factor;
COPD,: Chronic obstructive pulmonary disease;
WC,: Waist circumference;
ECG,: Electrocardiogram.
