# Supplementary material for: Respiratory rate and its associations with disease and lifestyle factors in the general population – results from the KORA-FF4 study
Source: PLoS One. 2025 Mar 11;20(3):e0318502. doi: 10.1371/journal.pone.0318502 (PMC11896064; doi:10.1371/journal.pone.0318502)
Supplement: S1 File — (PDF) [file pone.0318502.s001.pdf]

Supporting information

S1 Table. Specific cutoff values for the OGTT and further information used to define normal glucose regulation, pre-diabetes, and diabetes.

**No diabetes**	Normal glucose regulation	fasting glucose < 110 mg/dl AND<br>2-hour glucose < 140 mg/dl
**Pre-diabetes**	i-IFG - Isolated impaired fasting glucose	110 mg/dl ≤ fasting glucose < 126 mg/dl AND<br>2-hour glucose < 140mg/dl
	i-IGT- Isolated impaired glucose tolerance	fasting glucose < 110 mg/dl AND<br>140 mg/dl ≤ 2-hour glucose < 200 mg/dl
	IFG+IGT	110 mg/dl ≤ fasting glucose < 126 mg/dl AND<br>140 mg/dl ≤ 2-hour glucose < 200 mg/dl
**Diabetes**	Newly diagnosed diabetes by OGTT	fasting glucose ≥ 126 mg/dl OR<br>2-hour glucose ≥ 200 mg/dl
	Known diabetes	validated by physician or taking antidiabetic medication
**Unclear**	Unclear diabetes status, no validation possible	unclear self-reported diabetes status, no clear medication use, missing OGTT

S2 Table. Characteristics of the study population stratified by respiratory rate.

		< 18.6 brpm<br>N = 1,916 (86.2%)	≥ 18.6 brpm (elevated)<br>N = 308 (13.8%)
**Sex**	Women	1001 (52.2)	154 (50.0)
	Men	915 (47.8)	154 (50.0)
**Age**	39-48 years	417 (21.8)	55 (17.9)
	49-58 years	490 (25.6)	52 (16.9)
	59-68 years	478 (24.9)	78 (25.3)
	69-78 years	383 (20.0)	74 (24.0)
	79-88 years	148 (7.7)	49 (15.9)
**School education**	Highest	519 (27.2)	50 (16.2)
	Middle	509 (26.6)	63 (20.5)
	Lowest	883 (46.2)	195 (63.3)
**Living alone**	Lives with partner	1500 (78.3)	216 (70.1)
	Lives alone	416 (21.7)	92 (29.9)
**Waist circumference**	≤ 88cm in women, ≤ 102cm in men	987 (51.5%)	86 (27.9%)
	> 88cm in women, > 102cm in men	928 (48.4%)	220 (71.4%)
**Physical activity**	Active	1134 (59.2)	139 (45.1)
	Inactive	782 (40.8)	169 (54.9)
**Smoking**	Never-smoker	807 (42.1)	119 (38.6)
	Ex-smoker	826 (43.1)	130 (42.2)
	Smoker	283 (14.8)	59 (19.2)
**Alcohol intake**	No consumption	507 (26.5)	100 (32.5)
	Less than 40g/day	1182 (61.7)	170 (55.2)
	40g/day or more	226 (11.8)	38 (12.3)
**Hypertension**	No current hypertension	1201 (62.8)	154 (50.2)
	Current hypertension	711 (37.2)	153 (49.8)
**Myocardial infarction**	No myocardial infarction	1853 (97.0)	292 (94.8)
	Myocardial infarction	58 (3.0)	16 (5.2)
**Stroke**	No stroke	1874 (97.9)	290 (94.8)
	Stroke	41 (2.1)	16 (5.2)
**Cancer**	Never cancer	1705 (89.0)	271 (88.0)
	Cancer	211 (11.0)	37 (12.0)
**Depressive symptoms**	No/little depressive symptoms	1840 (96.1)	286 (93.2)
	Depressive symptoms	74 (3.9)	21 (6.8)
**COPD**	No COPD	1786 (93.5)	263 (85.9)
	COPD	124 (6.5)	43 (14.1)
**Asthma**	No asthma	1743 (91.1)	279 (91.2)
	Asthma	171 (8.9)	27 (8.8)
**Diabetes mellitus (OGTT)**	Normal glucose tolerance	1287 (67.2)	142 (46.1)
	Prediabetes	319 (16.6)	70 (22.7)
	Known or new diabetes	239 (12.5)	80 (26.0)
	Unclear	71 (3.7)	16 (5.2)
**Heart rate**	Mean (SD)	64.4 (8.9)	74.1 (12.4)
	Median (IQR)	64.2 (11.4)	73.9 (16.1)

COPD: chronic obstructive pulmonary disease; OGTT: results of the oral glucose tolerance test

S3 Table. Heart rate by age and sex.

**Age**	**Sex**	**N**	**Min**	**Max**	**Median**	**IQR**	**quantile 5**	**quantile 25**	**quantile 75**	**quantile 95**
39 - 48 y.	Men	221	45.11	98.68	**63.31**	12.13	50.49	57.26	69.39	82.27
	Women	251	48.57	92.76	**65.25**	10.59	52.43	60.35	70.95	80.25
49 - 58 y	Men	254	39.37	95.18	**63.84**	13.20	48.27	58.10	71.29	81.93
	Women	288	48.84	90.80	**64.82**	10.60	54.24	59.90	70.49	81.56
59 - 68 y.	Men	254	36.82	120.33	**63.46**	13.20	48.31	56.81	70.01	83.81
	Women	302	41.78	119.04	**66.33**	12.29	52.23	60.26	72.55	81.76
69 - 78 y.	Men	234	40.80	97.93	**64.79**	16.65	50.91	57.22	73.86	88.26
	Women	223	46.15	111.74	**67.36**	11.26	50.66	61.27	72.52	83.73
79 - 88 y.	Men	106	45.63	103.31	**64.33**	12.86	49.66	57.60	70.45	80.17
	Women	91	46.54	90.59	**66.19**	12.18	51.10	59.37	71.55	87.61

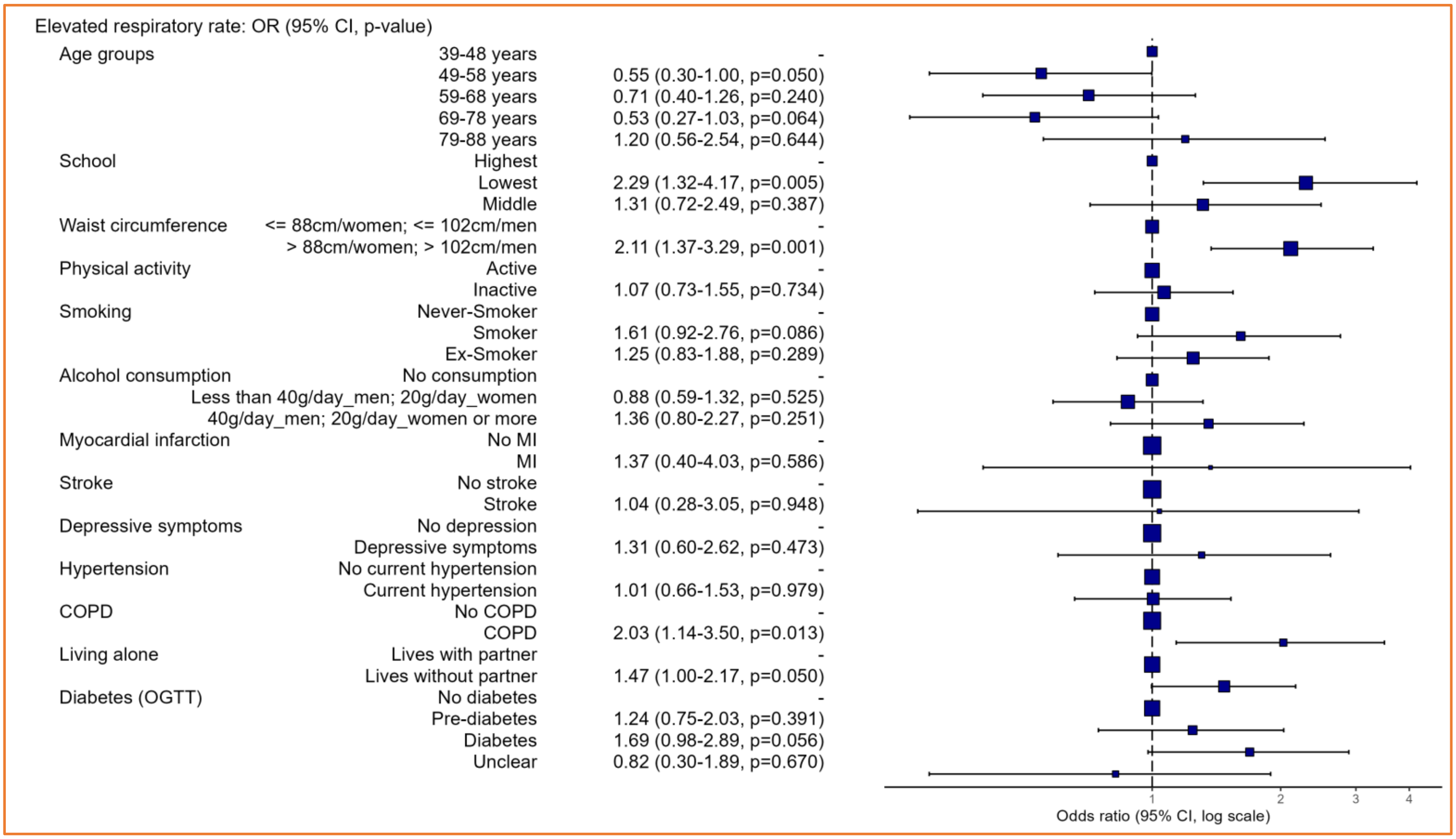

S1 Fig. Multivariable logistic regression model with the outcome elevated respiratory rate in women (N = 1,143 women with complete data).

Further model information: Number in data frame = 1155, Number in model = 1143, Missing = 12, AIC = 858.4, C-statistic = 0.709, H&L = Chi-sq(8) 5.86 (p=0.663)

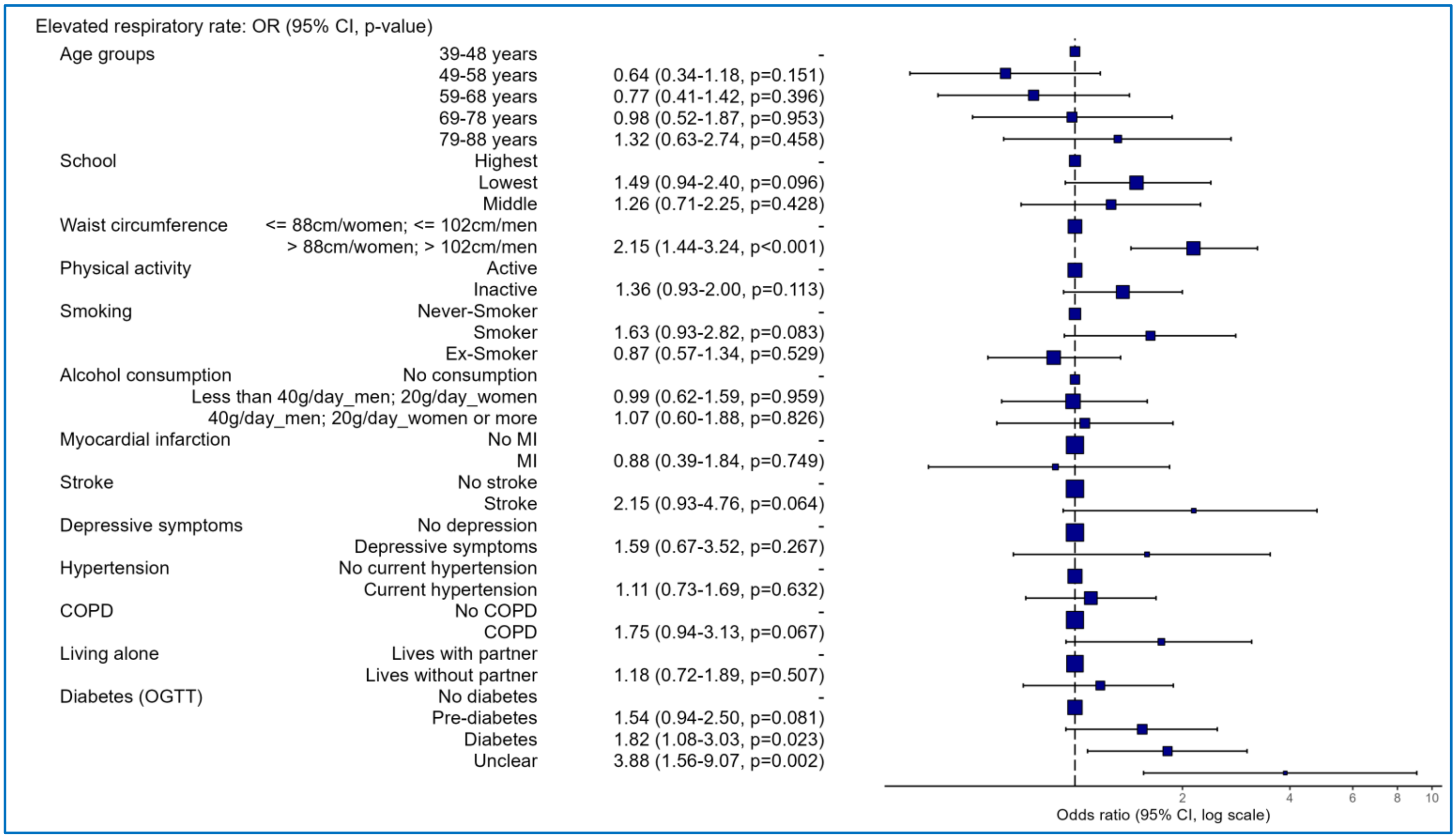

S2 Fig. Multivariable logistic regression model with the outcome elevated respiratory rate in men (N = 1,051 men with complete data).

Further model information: Number in data frame = 1069, Number in model = 1051, Missing = 18, AIC = 830, C-statistic = 0.718, H&L = Chi-sq(8) 5.53 (p=0.699)

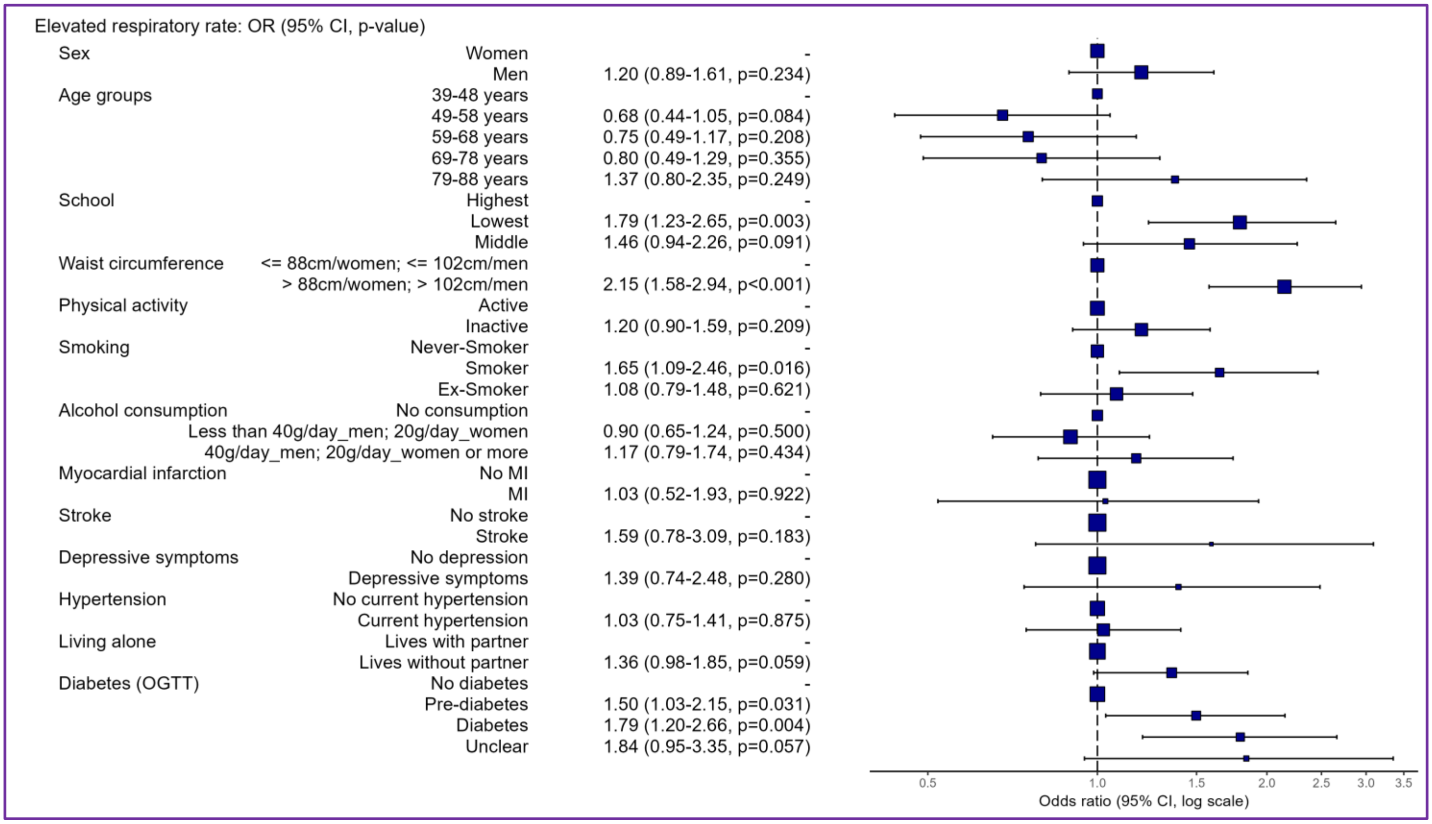

S3 Fig. Sensitivity Analysis: Multivariable logistic regression model excluding 167 participants with COPD - Association of various disease and lifestyle factors with elevated respiratory rate (N = 2,030 participants with complete data).

Further model information: Number in data frame = 2049, Number in model = 2030, Missing = 19, AIC = 1484.1, C-statistic = 0.699, H&L = Chi-sq(8) 3.94 (p=0.862)

Further model information for Figure 4:

Fig 4. Multivariable logistic regression – Association of various disease and lifestyle factors with elevated respiratory rate (N = 2,194 participants with complete data).

Further model information: Number in data frame = 2224, Number in model = 2194, Missing = 30, AIC = 1662.9, C-statistic = 0.704, H&L = Chi-sq(8) 2.35 (p=0.968)
